# Comparative metagenomics of the gut microbiota in wild greylag geese (*Anser anser*) and ruddy shelducks (*Tadorna ferruginea*)

**DOI:** 10.1002/mbo3.725

**Published:** 2018-09-17

**Authors:** Wen Wang, Sisi Zheng, Laixing Li, Yongsheng Yang, Yingbao Liu, Aizhen Wang, Kirill Sharshov, Yao Li

**Affiliations:** ^1^ State Key Laboratory of Plateau Ecology and Agriculture Qinghai University Xi'ning Qinghai China; ^2^ Northwest Institute of Plateau Biology Chinese Academy of Sciences Xi'ning Qinghai China; ^3^ College of Life Science Yangtze University Jingzhou Hubei China; ^4^ College of Eco‐Environmental Engineering Qinghai University Xi'ning Qinghai China; ^5^ Research Institute of Experimental and Clinical Medicine Novosibirsk Russia

**Keywords:** antibiotic resistance genes, carbohydrate‐active enzymes, greylag geese, gut metagenomes, ruddy shelducks

## Abstract

Gut microbiome contributes to host health by maintaining homeostasis, increasing digestive efficiency, and facilitating the development of immune system. Wild greylag geese (*Anser anser*) and ruddy shelducks (*Tadorna ferruginea*), migrating along the central Asian flyway, appear to be one of the most popular species in the rare birds rearing industries of China. However, the structure and function of the gut microbial communities associated with these two bird species remain poorly understood. Here, for the first time, we compared gut metagenomes from greylag geese to ruddy shelducks and investigated the similarities and differences between these two bird species in detail. Taxonomic classifications revealed the top three bacterial phyla, *Firmicutes*,* Proteobacteria,* and *Fusobacteria*, in both greylag geese and ruddy shelducks. Furthermore, between the two species, 12 bacterial genera were found to be more abundant in ruddy shelducks and 41 genera were significantly higher in greylag geese. A total of 613 genera (approximately 70%) were found to be present in both groups. Metabolic categories related to carbohydrate metabolism, metabolism of cofactors and vitamins, lipid metabolism, amino acid metabolism, and glycan biosynthesis and metabolism were significantly more abundant in ruddy shelducks, while greylag geese were enriched in nucleotide metabolism and energy metabolism. The herbivorous greylag geese gut microbiota harbored more carbohydrate‐active enzymes than omnivorous ruddy shelducks. In our study, a range of antibiotic resistance categories were also identified in the gut microbiota of greylag geese and ruddy shelducks. In addition to providing a better understanding of the composition and function of wild birds gut microbiome, this comparative study provides reference values of the artificial domestication of these birds.

## 1 INTRODUCTION

Animals are colonized by rich and complex communities of microorganisms, both externally (e.g., on skin and feathers) and internally (e.g., in the gastrointestinal and reproductive tracts) (Colston & Jackson, 2016; McFall‐Ngai et al., 2013). Advances in next‐generation sequencing and bioinformatic technologies permit the study of these microorganisms, their genes, and their metabolites (termed the microbiome) at an unprecedented scale (Eisen, 2015; Jovel et al., 2016). Trillions of microbes inhabit the gastrointestinal tract of animals, forming a dynamic ecological community within the gut, which is termed the “gut microbiome” (Ley et al., 2008; Lloyd‐Price, Abu‐Ali, & Huttenhower, 2016). A wealth of studies have shown that gut microbiome plays an important role in several fundamental and crucial processes in humans and other animal hosts, such as development (Malmuthuge, Griebel, & Guan, 2015), immune homeostasis (Ahern, Faith, & Gordon, 2014), nutrient assimilation (Kau, Ahern, Griffin, Goodman, & Gordon, 2011), vitamins synthesis and sterols metabolism (O'Mahony, Clarke, Borre, Dinan, & Cryan, 2015), and diseases (e.g., obesity, diabetes, and cancer) (Kinross, Darzi, & Nicholson, 2011; Lee & Hase, 2014). Given these important findings, many gut microbiome projects have been launched in several countries (Pylro, Mui, Rodrigues, Andreote, & Roesch, 2016; Stulberg et al., 2016). However, most of these projects and studies describe the microbiota of humans and some mammalian animals; a major gap identified was that there was no project for the vast majority of ecologically relevant taxa, birds.

Compared to other mammalian vertebrates, several characteristics make birds some of the most interesting and useful models for studying the gut microbiome. First, bird brood parasites lay their eggs in the nests of appropriate brood hosts, thus offering a unique and powerful model to investigate the influence of genetic and environmental factors on the colonizing process of gut microbiota (Hird, Carstens, Cardiff, Dittmann, & Brumfield, 2014). Second, whereas mammals acquire important maternal microbes during the birth process, many birds regurgitate food to their young, providing a mode of vertical transmission of gut microbiome across generations (Godoy‐Vitorino et al., 2010; Putignani, Del Chierico, Petrucca, Vernocchi, & Dallapiccola, 2014). Third, birds possess a cloaca, serving dual functions for excretion and sexual copulation. Thus, the gastrointestinal tract microbiota of birds provide another avenue for exploring the potential exchange of components of the endogenous microbiome during reproduction (Kreisinger, Cizkova, Kropackova, & Albrecht, 2015).

Currently, there are two main strategies for the analysis of gut microbiome using next‐generation sequencing, shotgun metagenomics, and 16S rRNA gene sequencing. The metagenomics approach, in which all the DNA fragments in a sample are sequenced rather than only 16S rRNA amplicons, results in greater in‐depth coverage and more informative sequencing datasets (Turaev & Rattei, 2016). Analyses of these datasets will help to elucidate the composition of microbial communities and are valuable resources for identifying carbohydrate‐active enzymes and antibiotics resistance genes present in gut communities. To the best of our knowledge, only very limited metagenomic analyses of the functional aspects of avian gut microbiota have been reported (Danzeisen, Kim, Isaacson, Tu, & Johnson, 2011; Godoy‐Vitorino et al., 2012; Lu, Santo Domingo, & Shanks, 2007; Wang, Zheng, et al., 2017; Wang, Song, et al., 2017). A meta‐analysis of these studies showed that the gut microbiota of birds were dominated by four major phyla, *Firmicutes*,* Proteobacteria*,* Actinobacteria,* and *Bacteroidetes* (Waite & Taylor, 2014, 2015).

In this study, metagenomic sequencing was performed to compare the gut microbial compositions and functions of two bird species, greylag geese (*Anser anser*) and ruddy shelducks (*Tadorna ferruginea*). These birds belong to the same family (Anatidae) and are two ecologically and economically important waterfowl. greylag geese and ruddy shelducks have worldwide distributions and migrate along the central Asian flyway between their breeding and wintering areas (Takekawa et al., 2013). In addition, these two species are artificially reared in several provinces of China to meet market demands (e.g., meat and eggs) and for conservation purposes. The results of this study provide a deeper exploration of the gut microbiomes of wild geese and ducks and may provide useful information for the further application of probiotic strains (isolated from the feces of wild geese and ducks) in the artificial rearing of these birds.

## 2 MATERIALS AND METHODS

### 2.1 Ethics statement

This study conformed to the guidelines for the care and use of experimental animals established by the Ministry of Science and Technology of the People's Republic of China (Approval number: 2006‐398). The research protocol was reviewed and approved by the Ethical Committee of Qinghai University. In this study, only feces of greylag geese and ruddy shelducks were collected for relevant molecular studies. No direct capture or hunting involved.

### 2.2 Sampling

Fecal samples were taken from three wild greylag geese (in text abbreviation: GG group) and three wild ruddy shelducks (in text abbreviation: RSD group) from the Gengga‐hai Lake (N36°11′59.8″ E100°05′39.7″, elevation 2,800 m) on the northeastern Qinghai‐Tibet Plateau, Qinghai Province, China. We chose the sampling sites utilized by these birds as stopover in autumn during migration and waited when they were foraging in the farmlands where only a single species was present in the morning. Fresh fecal material was collected and stored in sterile tubes. The fecal samples were collected at a minimum distance interval of five meters to ensure that all fresh droppings were expelled from different individuals. All samples were transported to the laboratory using a −20°C portable freezer and stored at −80°C until further treatment.

### 2.3 DNA extraction and metagenomic sequencing

Metagenomic DNA was isolated from approximately 1 g of fecal sample, using the E.Z.N.A.^®^ stool DNA Kit (Omega Bio‐tek, Norcross, GA, USA) following the manufacturer's instruction. DNA concentration and quality were assessed by Qubit fluorometer and agarose gel electrophoresis, respectively. The high‐quality DNA was then used to create an Illumina DNA library and sequenced using Illumina NovaSeq (2 × 125 bp) (Illumina, USA) platform at Novogene Bioinformatics Technology Co. Ltd (Beijing, China).

### 2.4 Sequence analyses and metagenome assembly

To obtain the clean data for subsequent analysis, the raw data from the Illumina NovaSeq sequencing platform were processed using Readfq (V8, https://github.com/cjfields/readfq). The specific processing steps were as follows: (a) removed reads which contain low‐quality bases (default quality threshold value less than or equal to 38) above a certain portion (default length of 40 bp); (b) removed reads in which the N base reached a certain percentage (default length of 10 bp); (c) removed reads which shared the overlap above a certain portion with Adapter (default length of 15 bp). To filter the reads that were of host origin, clean data were then blast against the host database using SoapAligner software (soap2.21, http://soap.genomics.org.cn/soapaligner.html). The parameters were as follows: identity greater than or equal to 90%, ‐l 30, ‐v 7, ‐M 4, ‐m 200, ‐x 400 (Law et al., 2013). Then, the high‐quality reads of each sample were assembled by the SOAPdenovo software (V2.04, http://soap.genomics.org.cn/soapdenovo.html) (Luo et al., 2012), with the parameters ‐d 1, ‐M 3, ‐R, ‐u, ‐F, ‐K 55 (Qin et al., 2014). After de novo assembly for each sample independently, all reads that not used from all samples were combined and performed mixed assembly in order to maximize the usage of data. Subsequently, we broke the assembled Scaffolds from N connection and obtained the Scaftigs. At last, the fragments longer than 500 bp in all of Scaftigs were used for further analysis.

### 2.5 Gene prediction and construction of the nonredundant gene set

We used MetaGeneMark (V2.10, http://topaz.gatech.edu/GeneMark/) to predict ORFs from the Scaftigs assembled from each sample as well as the Scaftigs from the mixed assembly. Then, the ORFs with length <100 bp were filtered out. For the predicted ORFs, CD‐HIT software (V4.5.8, http://www.bioinformatics.org/cd-hit) was used to reduce sequence redundancy (Fu, Niu, Zhu, Wu, & Li, 2012) and the unique initial gene catalogue (the genes here refer to the nucleotide sequences coded by unique and continuous genes) was obtained (Sunagawa et al., 2015). To obtain the gene catalogue (Unigenes) eventually used for subsequently analysis, the clean data of each sample were mapped to the unique initial gene catalogue using SoapAligner (soap 2.21). Based on the number of mapped reads and the length of gene, the abundance information of each Unigene in each sample was statistically analyzed.

### 2.6 Gene taxonomic prediction

DIAMOND software (V0.7.9, https://github.com/bbuchfink/diamond/) was used to blast the unigenes to the sequences of bacteria, fungi, archaea, and viruses which were all extracted from the NR database (version: 20161115, https://www.ncbi.nlm.nih.gov/) of NCBI with the parameter of blastp, ‐e 1e‐5 (Buchfink, Xie, & Huson, 2015). To identify bacterial taxa, the lowest common ancestor (LCA) algorithm in MEGAN software was used (Huson, Mitra, Ruscheweyh, Weber, & Schuster, 2011). The exhibition of PCA (R ade4 package, version 2.15.3) (Avershina, Trine, & Knut, 2013) and NMDS (R vegan package, version 2.15.3) (Rivas et al., 2013) decrease‐dimension analyses was based on the abundance of each taxonomic hierarchy. Analysis of similarities (ANOSIM) was performed using the vegan package in R (version 2.15.3). Metastats analyses were used to look for the different species between groups. Venn diagram analyses at the genus level were performed using VennDiagram package in the software R (2.15.3).

### 2.7 Functional gene annotation

Functional annotation of metagenomes was conducted using DIAMOND software (V0.7.9) to blast unigenes to KEGG database (version 201609, http://www.kegg.jp/kegg/) (Kanehisa et al., 2014), CAZy database (version 20150704, http://www.cazy.org/) (Cantarel et al., 2009), and the Comprehensive Antibiotic Resistance Database (https://card.mcmaster.ca/) with the parameter setting of blastp, evalue ≤ 1e ‐ 30 (Jia et al., 2017). For each sequence's blast result, the best Blast Hit was used for subsequent analysis. The relative abundance of each functional hierarchy equaled the sum of relative abundance annotated to that functional level. Based on the abundance of each hierarchy, heat map of hierarchy cluster and Metastats analysis were performed. Permutation test between groups was used in Metastats analysis, and the *p* value was corrected by controlling the false discovery rate using the Benjamini–Hochberg method (White, Nagarajan, & Pop, 2009).

## 3 RESULTS

### 3.1 Summary of the metagenomic datasets

A total of six metagenomes were sequenced using an Illumina's NovaSeq platform (2 × 150 bp). The output data encompassed a total of 38,306.65 Mb of raw reads, with an average of 6,384.44 Mb per sample (Supporting Information Table S1). Then, these sequenced raw reads were filtered to obtain clean reads by removing the short and low‐quality reads, adaptors and any eukaryotic sequences. As a result, a total of 36,473.30 Mb of clean reads were generated (Supporting Information Table S1) for further clear assembly and annotation analyses. The de novo assembly of these clean reads resulted in a total of 320.93 Mb of scaftigs (with the total length ranging from 14.49 to 117.78 Mb), with an N50 of 1150.67 bp (Supporting Information TableS1). Based on these scaftigs, a total of 310,560 unigenes with an average length of 550.27 bp and an average GC content of 42.93% were predicted using the gene‐finding algorithm MetaGeneMark (Supporting Information Table S1). These unigenes were then used for taxonomic analysis and functional annotation, and the results of which were summarized in Supporting Information Table S2.

### 3.2 Comparison of the composition of the metagenome‐based microbial communities in greylag geese and ruddy shelducks

**Figure 1:**
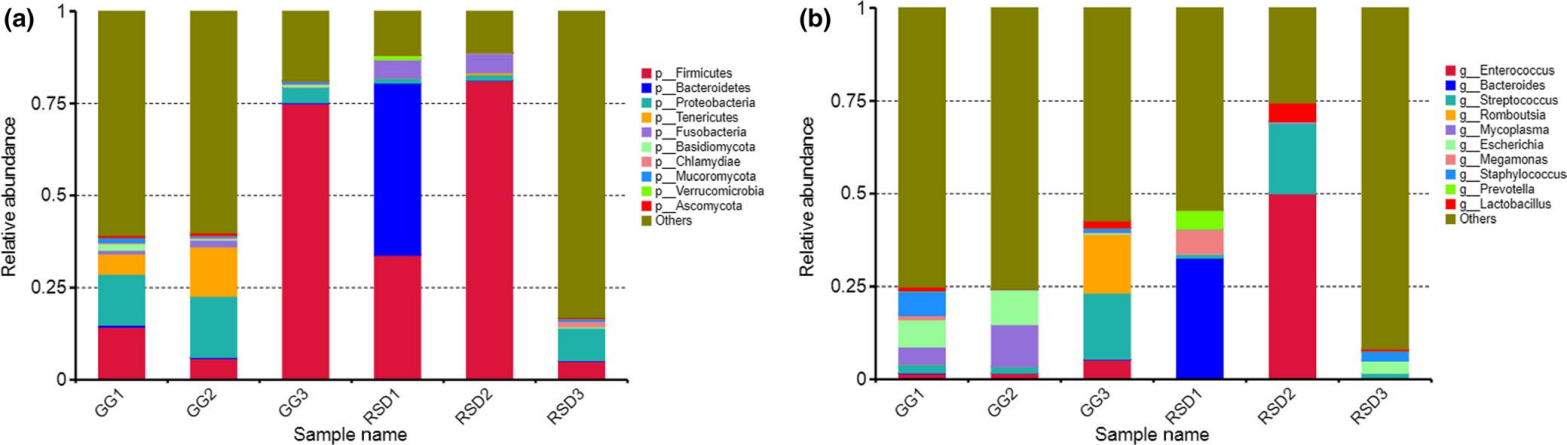
Taxonomic profiles of the microbial communities at the phylum level (a) and genus level (b) in each sample. GG refers to the greylag geese group; RSD refers to the ruddy shelducks group

To investigate the gut microbiome compositions of both greylag geese and ruddy shelducks, based on the BLAST results, approximately 150,882.57 unigenes were further classified into phyla and down to species level using the MEGAN software (Supporting Information Table S2). The top four phyla in greylag geese were *Firmicutes* (31.64%), *Proteobacteria* (11.51%), *Tenericutes* (6.34%), and *Fusobacteria* (0.96%) (Figure 1a and Supporting Information Table S3). In the ruddy shelducks group, *Firmicutes* also held the overwhelming predominance, with the average relative abundance of 39.99%, followed by *Bacteroidetes* (15.66%), *Proteobacteria* (3.93%), and *Fusobacteria* (3.40%) (Figure 1a and Supporting Information Table S3). Sequences that could not be classified into any known groups and that were detected with low abundance were grouped as “others.” The proportion of these unclassified sequences varied between 11.19% and 83.09% among the different samples (Figure 1a and Supporting Information Table S3). This indicated that a great number of unknown bacteria were present in the guts of these two species of birds. The most abundant genus was *Streptococcus* (7.25%), followed by *Escherichia* (5.72%), *Mycoplasma* (5.34%), *Romboutsia* (5.27%), and *Staphylococcus* (2.60%) in greylag geese (Figure 1b and Supporting Information Table S4). In contrast, the dominant microbial genera in ruddy shelducks were *Enterococcus* (16.70%), *Bacteroides* (10.87%), *Streptococcus* (7.17%), *Megamonas* (2.22%), and *Lactobacillus* (1.85) (Figure 1b and Supporting Information Table S4).

**Figure 2:**
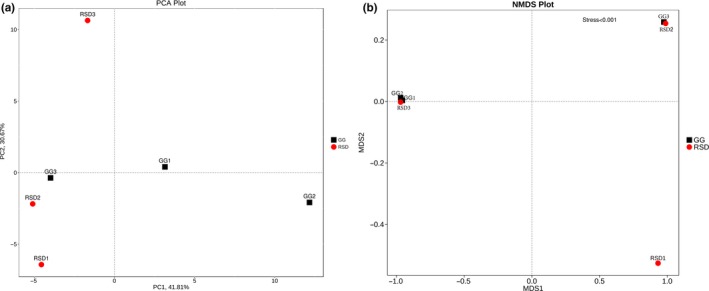
The beta diversity results of PCA plot (a) and NMDS plot (b) indicating the microbial phyla distribution between the groups. GG refers to the greylag geese group samples; RSD refers to the ruddy shelducks group samples

Differences in bacterial community composition between the two groups were estimated using principal component analysis (PCA) and nonmetric multidimensional scaling (NMDS) (Figure 2). At the phylum level, greylag geese populations were observed to demonstrate a visually distinct profile from the ruddy shelducks. These dissimilarities in microbial communities were further confirmed by performing genus‐level PCA and NMDS calculations (Supporting Information Figure S1). Furthermore, analysis of similarities (ANOSIM) was performed to determine whether the total variation in the gut microbiome was due to the differences within or between groups. The results showed that differences observed within group outweighed that occurring between groups (Supporting Information Figure S2). These interindividual variances may be partially due to the unequal and relatively small sample size of this study.

**Figure 3:**
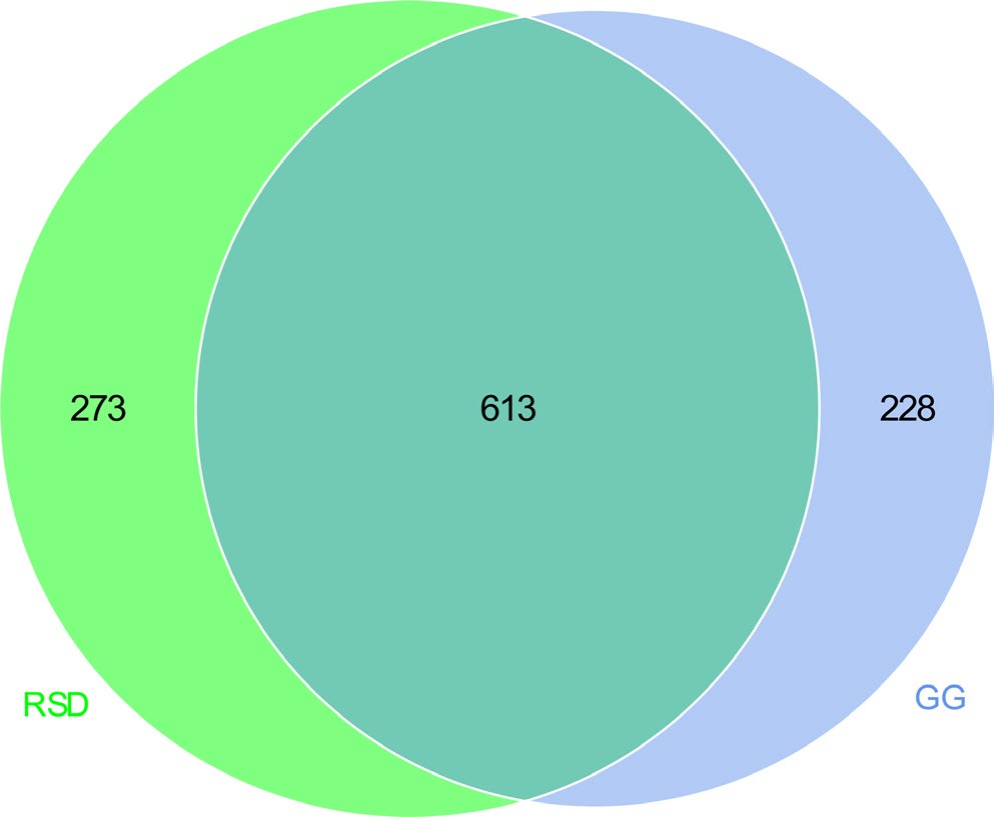
Venn diagrams showing the unique and shared microbial genera between Group GG and Group RSD. GG refers to the greylag geese group; RSD refers to the ruddy shelducks group

Metastats analyses were performed to detect differentially abundant genera between the two groups (Supporting Information Table S5). A total of 12 genera were found to be more abundant in RSD group and 41 genera were significantly higher in GG group (Supporting Information Table S5). A Venn diagram showed that 613 genera were assigned to both groups, while 273 and 228 bacterial genera were only assigned to the RSD and GG groups, respectively (Figure 3).

### 3.3 Functional profiling of the gut metagenome

**Figure 4:**
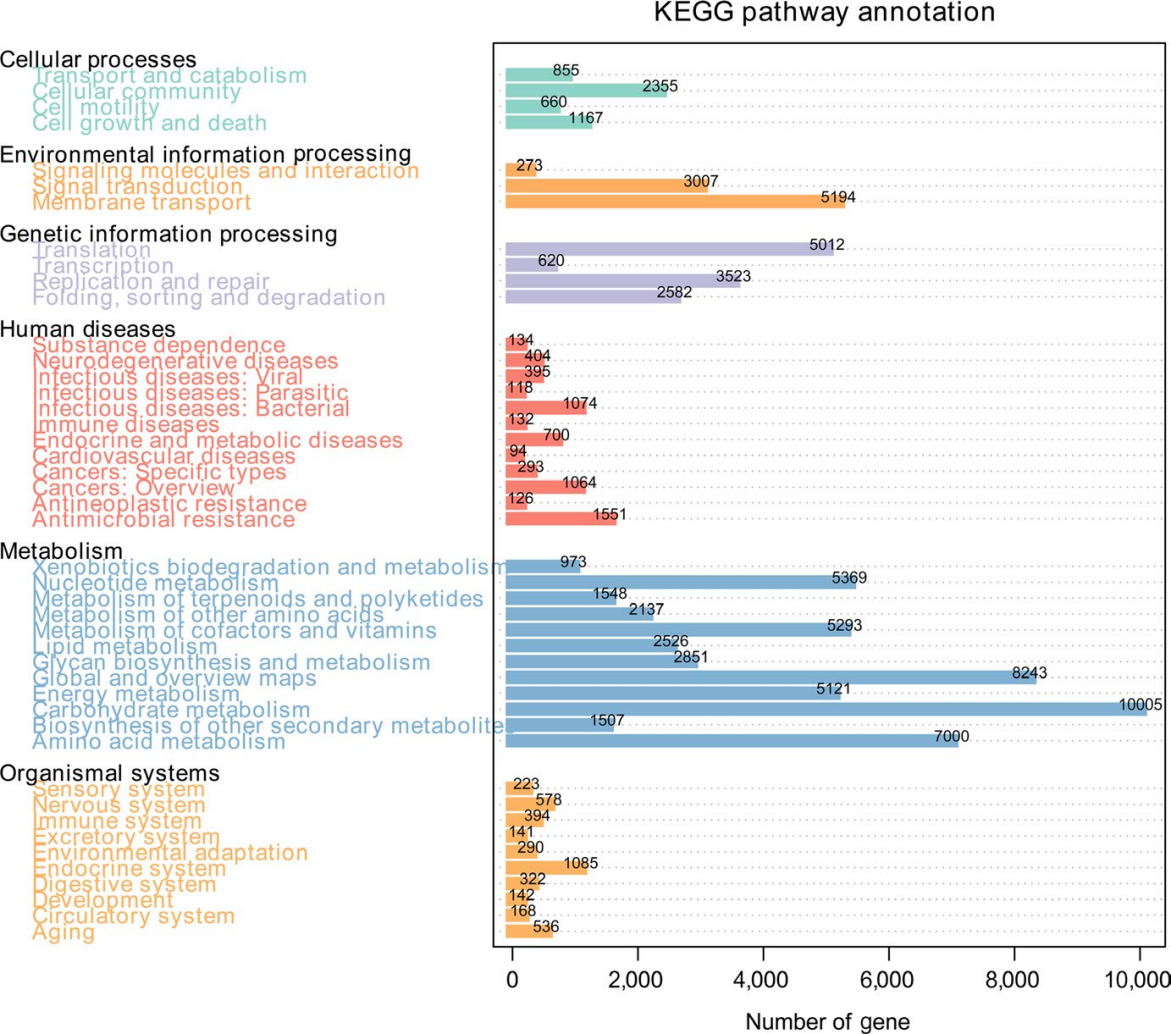
Summary of unigenes matched to each KEGG functional categories (level 1 and level 2) present in the gut metagenome datasets

**Figure 5:**
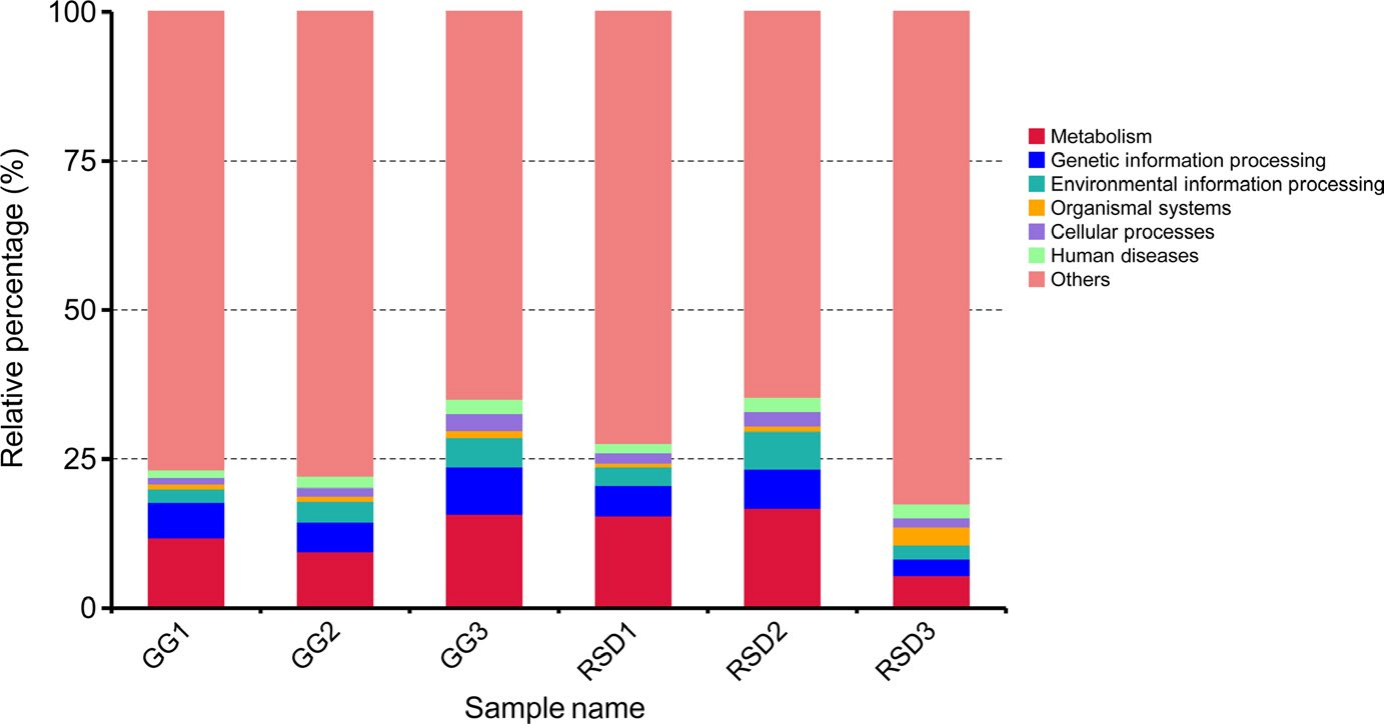
Relative abundance of different KEGG functional categories present in each sample. GG refers to the greylag geese group; RSD refers to the ruddy shelducks group

Metagenome sequencing has the inherent advantage of allowing for an examination of gene content of the microbial populations and allows the direct inference of the metabolic capacity of these populations. To explore the overall functional profiles of the gut microbiome, a total of 141,279 out of 310,560 unigenes (45.49%) were identified by the KEGG database (Supporting Information Table S2). Of these unigenes, 82,371 (26.52%) could be assigned to 7,021 KEGG ortholog group (KOs) and 49,795 (16.03%) could be assigned to 384 KEGG pathways (Supporting Information Table S2). The unigenes matching to level 1 and level 2 KEGG functional categories were shown in Figure 4. In detail, the dominant functional categories identified included metabolism (59.89%), genetic information processing (13.37%), and environmental information processing (9.65%). The cumulative proportion of these three categories was above 82.91% for all assigned unigenes. This indicated that the metabolic potential of the gut microbiota related to these two species of birds was highly active. The proportions of KEGG categories (level 1) in each sample were shown in Figure 5.

### 3.4 Comparison of functionality of the greylag geese and ruddy shelducks gut metagenomes

**Figure 6:**
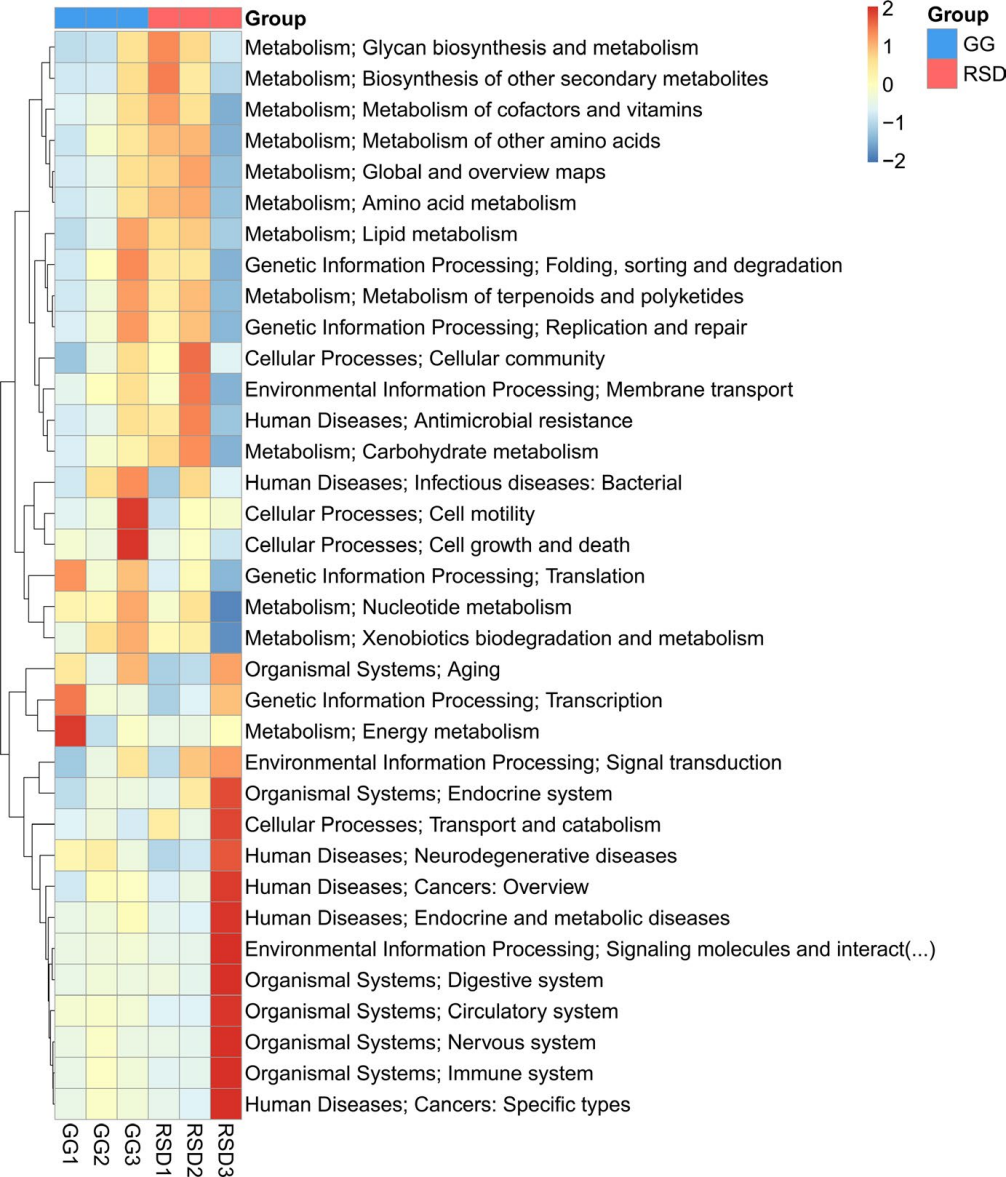
Heat map of hierarchical clustering analysis of the top 35 abundant KEGG functional categories (level 2) in Group GG and Group RSD. GG refers to the greylag geese group; RSD refers to the ruddy shelducks group

Due to differences in genetics and diet compositions, significantly different abundances of functional genes and pathways were expected to be observed in greylag geese and ruddy shelducks. A heat map of a hierarchical clustering analysis of the top 35 abundant KEGG functional categories (level 2) showed that increased metabolic categories involved in carbohydrate metabolism, metabolism of cofactors and vitamins, lipid metabolism, amino acid metabolism, and glycan biosynthesis and metabolism in RSD group, whereas the GG group were enriched in nucleotide metabolism and energy metabolism (Figure 6). Furthermore, among the detected 384 KEGG pathways (level 3), nine pathways were found to be significantly different between GG and RSD groups through a Metastats analysis (Supporting Information Table S6).

### 3.5 Diversity profile of CAZymes

Carbohydrate‐active enzymes (CAZymes), encoded by gut microbes, play a crucial role in the breakdown of complex dietary carbohydrates into components that can be absorbed by the host intestinal epithelium. To determine the gut CAZymes profiles, we performed a CAZymes analysis using the metagenomic data from both greylag geese and ruddy shelducks. In total, 9,060 putative genes were identified (Supporting Information Table S2). The majority of these genes identified were assigned to glycoside hydrolases (5,491, 57.78%). In the RSD group, a total of 210 CAZymes were identified, including five auxiliary activities (AAs), 43 carbohydrate binding modules (CBMs), 12 carbohydrate esterases (CEs), 89 glycoside hydrolases (GHs), 52 glycosyl transferases (GTs), and nine polysaccharide lyases (PLs). A total of 196 CAZymes were found in the GG group, including five AAs, 38 CBMs, 10 CEs, 78 GHs, 58 GTs, and seven PLs. The proportions of each CAZymes were compared using Metastats analysis. GH24, 99, 104 and GT33, 72, 73, 80 were found to be significantly higher in GG group (*p* < 0.05) than that in RSD group, while three CAZymes had higher proportions in RSD group (*p* < 0.05), including GH27, GT10, and CBM20.

### 3.6 Antibiotic resistance profiles

**Figure 7:**
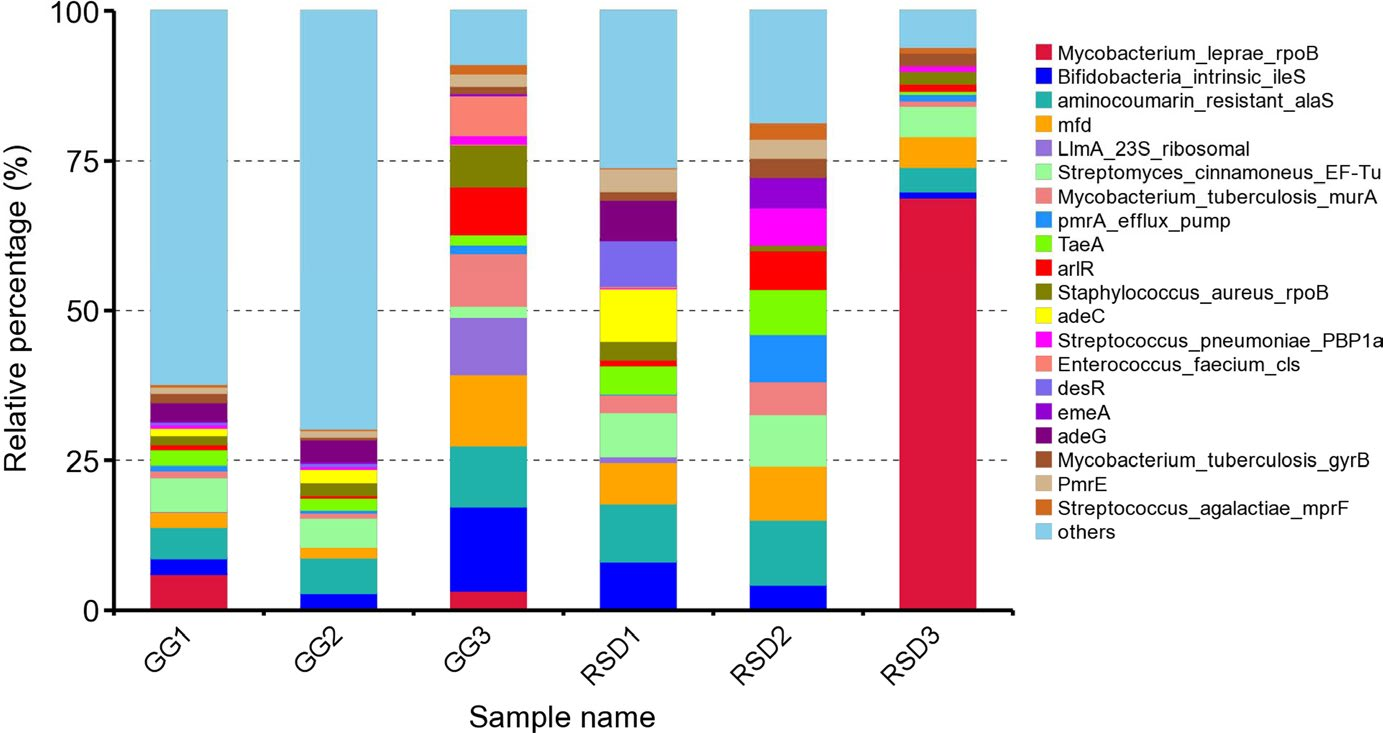
Relative proportion of the top 20 most abundant antibiotic resistance ontology in each sample. GG refers to the greylag geese group samples; RSD refers to the ruddy shelducks group samples

**Figure 8:**
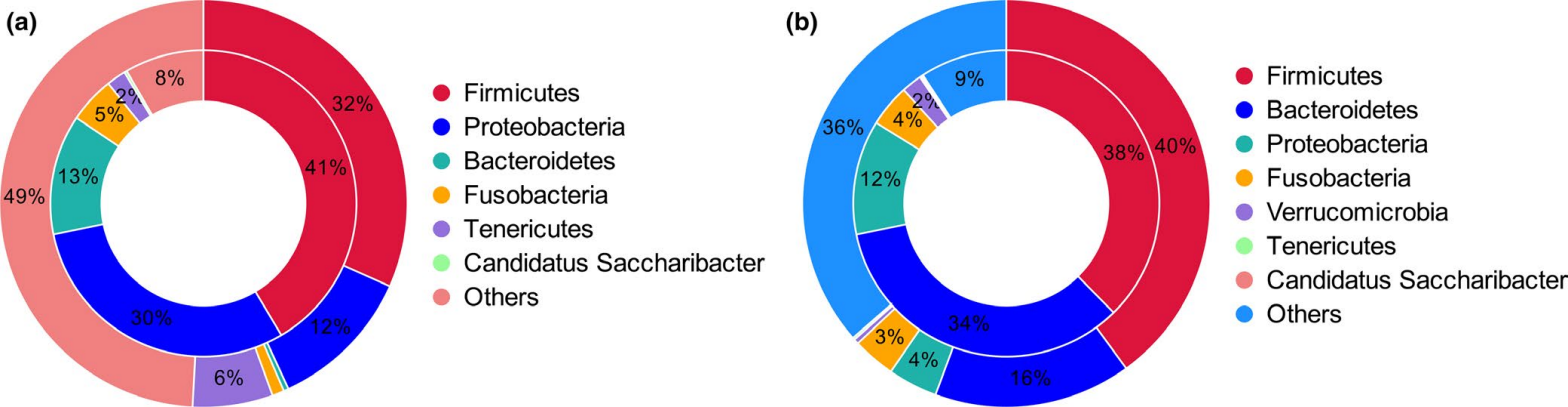
Circos plots representing alignment of the proportion of different antibiotic resistance ontology and microbial phyla in Group GG (a) and Group RSD (b). The inner‐ring refers to the distribution of different antibiotic resistance ontology in corresponding microbial phyla. The outer‐ring refers to the relative abundance of different phyla in each group. GG refers to the greylag geese group; RSD refers to the ruddy shelducks group

The high concentrations of antibiotics used in clinical, agricultural, livestock, and poultry settings provide a strong selective pressure that favors the exchange of antibiotic resistance genes (ARGs) between pathogens and gut microbes. To investigate the ARGs present in the gut microbiota of greylag geese and ruddy shelducks, the unigenes identified in the metagenome data were screened for antibiotic resistance factors using the Comprehensive Antibiotic Resistance Database (CARD). A total of 598 unigenes were annotated in CARD and 125 antibiotic resistance ontologies (AROs) were identified (Supporting Information Table S2). Of these detected AROs, the top 20 most abundant AROs in each sample were shown in Figure 7. A range of antibiotic resistance categories were also identified, including resistance to rifampin, mupirocin, novobiocin, pulvomycin, fosfomycin, norfloxacin, ciprofloxacin, acriflavine, pleuromutilin, amoxicillin, daptomycin, aminocoumarin, and polymyxin. These AROs were further analyzed for their microbial origin. In the GG group, approximately 50% of the AROs were highly enriched in phyla *Firmicutes* (32%), *Proteobacteria* (12%), and *Tenericutes* (6%) (Figure 8a). In the RSD group, approximately 60% of AROs were found to be enriched in phyla *Firmicutes* (40%), *Bacteroidetes* (16%), *Proteobacteria* (4%), and *Fusobacteria* (3%) (Figure 8b). These results indicate that different types of gut microbes contribute differently to the occurrence of antibiotic resistance genes.

## 4 DISCUSSION

The results presented in this study represent the first gut metagenomic characterizations of both greylag geese and ruddy shelducks. To date, although several datasets are available for the gut metagenomes of domestic birds (e.g., chickens and ducks) (Day, Ballard, Duke, Scheffler, & Zsak, 2010; Delforno et al., 2017; Lu et al., 2007), relatively little information is available concerning the metagenomes of wild birds’ gut microbiota (Godoy‐Vitorino et al., 2008, 2012; Wang, Zheng, et al., 2017; Wang, Song, et al., 2017). Therefore, this study provides a significant contribution to the vast, yet little explored, field of wild birds’ gut microbiome. Additionally, knowledge of the gut microbiomes of these two wild bird species could also provide a helpful reference for improving the rearing performance of wild birds in artificial rearing industries.

It was a logical choice to compare greylag geese samples with the ruddy shelducks’ gut microbiota, because these two species of birds inhabit the same region and share part of the same food items. Based on the taxonomic classification, the gut microbiotas of greylag geese and ruddy shelducks differed substantially at the phylum and lower taxonomic levels. The reasons for these differences were not clear. Maybe the different genetic backgrounds were responsible for these differences. Support for a host genetic effect on the gut microbiome comes mostly from large comparative studies. For instance, Hird et al. sampled 116 intestines from 59 neotropical bird species and found that host taxonomic categories were most frequently able to significantly explain the most variations in the gut microbiotas of birds (Hird, Sánchez, Carstens, & Brumfield, 2015). In mammals, such studies had also shown that host taxonomy was strongly associated with gut microbiotas (Ley et al., 2008). Additionally, our study identified some common bacterial microbiota present in both greylag geese and ruddy shelducks. At the phylum level, *Firmicutes*,* Proteobacteria,* and *Fusobacteria* were found to be present at high abundance in each sample. *Firmicutes* and *Proteobacteria*, the most widespread intestinal phyla, are commonly observed within gut environments of many birds (Waite & Taylor, 2014; Wang, Cao, Li, et al., 2016; Wang, Cao, Yang, et al., 2016; Wang, Zheng, et al., 2016). Members of these two phyla were frequently studied for their food digestion roles. For example, *Firmicutes* members were associated with insoluble fiber degradation (Berry, 2016), and *Proteobacteria* members were associated with cellulose activity (Reid, Addison, Macdonald, & Lloyd‐Jones, 2011). A rich community of *Fusobacteria* was frequently reported in the guts of carnivorous and omnivorous birds (Waite & Taylor, 2014). In our study, the guts of ruddy shelducks contained 3.4% *Fusobacteria*. As an omnivorous bird, ruddy shelducks feed mainly on fish, shrimps, crabs, aquatic plants, and cereals. The appearance of *Fusobacteria* in the greylag geese gut microbiome is an interesting avenue for further study, since this species was considered as an herbivorous bird, consuming a diversity of foods that includes leaves, roots, and seeds (Olsen, 2015). At the genus level, the results showed that about 70% of genera were assigned to both greylag geese and ruddy shelducks groups. These common genera may be shaped by the overlapping food items between two groups. Diet is a factor that directly affects gut microbial community composition (Zarrinpar, Chaix, Yooseph, & Panda, 2014). However, the fundamental characteristics of the relationship between the diets and the wild birds’ gut microbiome are unknown. It should be of great research and practical application values to analyze these common genera to develop probiotics that may meet the demands of the artificial rearing industries for greylag geese and ruddy shelducks, helping to accelerate the domestication of these birds.

An Analysis of the overall functional profiles in the present study indicated that the gut microbes associated with these two bird species exhibited high metabolic activities. These results were consistent with earlier studies on the gut metagenomes of bar‐headed geese (*Anser indicus*) (Wang, Zheng, et al., 2017; Wang, Song, et al., 2017). This high metabolic rate may be related to the energy consumption required to fulfill the demands of flight. Avian metabolism was reported to be approximately 60% higher than that those of most mammals (Scanes & Braun, 2012). The further comparison of the functional profiles of the datasets from greylag geese and ruddy shelducks revealed many remarkable differences, which suggested that not only the bacterial compositions but their functionalities were important. For example, functions related to carbohydrate metabolism, lipid metabolism, amino acid metabolism, and glycan biosynthesis and metabolism were significantly more abundant in ruddy shelducks than that in greylag geese. It is reasonable to hypothesize that the microbiota of the omnivorous ruddy shelducks is probably specialized to degrade more diverse types of foods than that of the herbivorous greylag geese, which eats a more homogenous type of food. Several KEGG pathways (level 3) with different abundance dynamics in ruddy shelducks vs. greylag geese appear to be associated with the acclimation of microbiota to the nutrients. For example, we observed increases in the abundance of pentose and glucuronate interconversions [ko00040] in ruddy shelducks that may contribute to host glucose metabolism. We also observed increases in the abundance of pathways associated with limonene and pinene degradation [ko00903] and carotenoid biosynthesis [ko00906] in greylag geese that may contribute to the degradation and fermentation of plant material.

From these metagenome data, we also identified 9,060 putative carbohydrate‐active genes. Conforming to expectations, the herbivorous greylag geese gut microbiota harbor more CAZymes than that of the omnivorous ruddy shelducks. These detected CAZymes would allow greylag geese and ruddy shelducks to make extensive use of plant material as a source of nutrients through the enzymatic activities of the gut microbes. The majority of the CAZymes genes identified were assigned to glycoside hydrolases in each sample. GHs are the most abundant enzymes used to break down polysaccharides into smaller products (Berlemont & Martiny, 2016). Of the total detected GHs, GH24 (predominant activity is lysozyme), GH99 (predominant activity is glycoprotein endo‐α‐1,2‐mannosidase), and GH104 (predominant activity is peptidoglycan lytic transglycosylase) were found to be significantly increased in greylag geese group, while the proportion of GH27 (predominant activity is α‐galactosidase) was significantly increased in ruddy shelducks group. These increased GHs in each group indicated an enrichment of different gut microbes that were specialized in utilization of diverse plant polysaccharides.

Due to unmonitored application of antibiotics, the widespread of antibiotic resistance genes (ARGs) and antibiotic resistant bacteria has become a great public concern (Wright, 2007). In our study, a range of antibiotic resistance categories were also identified in the gut microbiota of greylag geese and ruddy shelducks. The existence of these antibiotic resistance genes in wild birds supported the view that antibiotic resistance was naturally originated. Soil was increasingly recognized as a vast repository of antibiotic resistance genes (Forsberg et al., 2012). Consequently, it is difficult to find a bird that has never been exposed to antibiotic polluted environments. Based on the analysis of the origin of these antibiotic resistance genes, ARGs profiles were found to be correlated with microbial community compositions. For instance, the majority of ARGs in greylag geese originated from the dominant phyla *Firmicutes* and *Proteobacteria*, and in ruddy shelducks from the phyla *Firmicutes* and *Bacteroidetes*. These phyla, known as prevalent antibiotic producing bacteria, were also reported to be present in the gut microbiota of four songbird species (Carter et al., 2018). These results indicated that highly mobile wildlife, such as migratory birds, might enable the spread of antibiotic resistant bacteria when they are exposed to previously unexposed environments (Viana, Santamaría, & Figuerola, 2016). These results also indicated that manure management was important in wildfowl artificial rearing industries. Thus, it is necessary to reduce potential environmental contamination risks associated with antibiotic resistance from feces of these birds and the abuse of antibiotics.

The present study had several limitations that should be acknowledged. First, the relatively small sample size may reduce the accuracy of partial results. Second, because of feces used in this study were collected from wild birds, some physiological indexes of these birds remain unknown. Therefore, large variances existed between individuals. Lastly, large datasets under captive environments are needed to investigate potential mechanisms driving diets–microbiota interactions in the birds’ gut.

In summary, we used metagenomics to gain an insight into both the compositional (profiles of microbiota) and the functional capabilities (KEGG functional categories, carbohydrate‐active enzymes, and antibiotic resistance genes) of the gut microbiomes of two wild bird species, greylag geese and ruddy shelducks. By comparing the gut metagenomes of these two species, we also identified both substantial overlap and differences in microbial composition and function. Although it remains unclear to what extent these changes were determined by host genetics and/or diets, these results substantially increased our knowledge of the bird gut microbiome.

## AUTHOR CONTRIBUTIONS

Wen Wang and Laixing Li participated in the design of the study, data analysis, and drafted the manuscript; Sisi Zheng and Yongsheng Yang participated in data analysis; Yingbao Liu, Aizhen Wang, Kirill Sharshov, and Yao Li participated in the design of the study and helped draft the manuscript. All authors gave final approval for publication.

## CONFLICT OF INTEREST

The authors declare that they have no conflict of interest.

## Data Availability

All the raw metagenome sequences data reported in this paper have been deposited in the Genome Sequence Archive (Wang, Zheng, et al., 2017; Wang, Song, et al., 2017) in BIG Data Center (BIG Data Center Members, 2018), Beijing Institute of Genomics (BIG), Chinese Academy of Sciences, under accession numbers CRA000822 and CRA000823 that are publicly accessible at http://bigd.big.ac.cn/gsa.
