# Supplementary material for: Comparative metagenomics of the gut microbiota in wild greylag geese (Anser anser) and ruddy shelducks (Tadorna ferruginea)
Source: Microbiologyopen. 2018 Sep 17;8(5):e00725. doi: 10.1002/mbo3.725 (PMC6528571; doi:10.1002/mbo3.725)
Supplement: Supplementary file 1 [file MBO3-8-e00725-s001.docx]

**Supporting Information**

**FIGURE S1**. The beta diversity results of PCA plot (A) and NMDS plot (B) indicating the microbial genera distribution between the groups. GG refers to the Greylag geese group samples, RSD refers to the Ruddy shelducks group samples


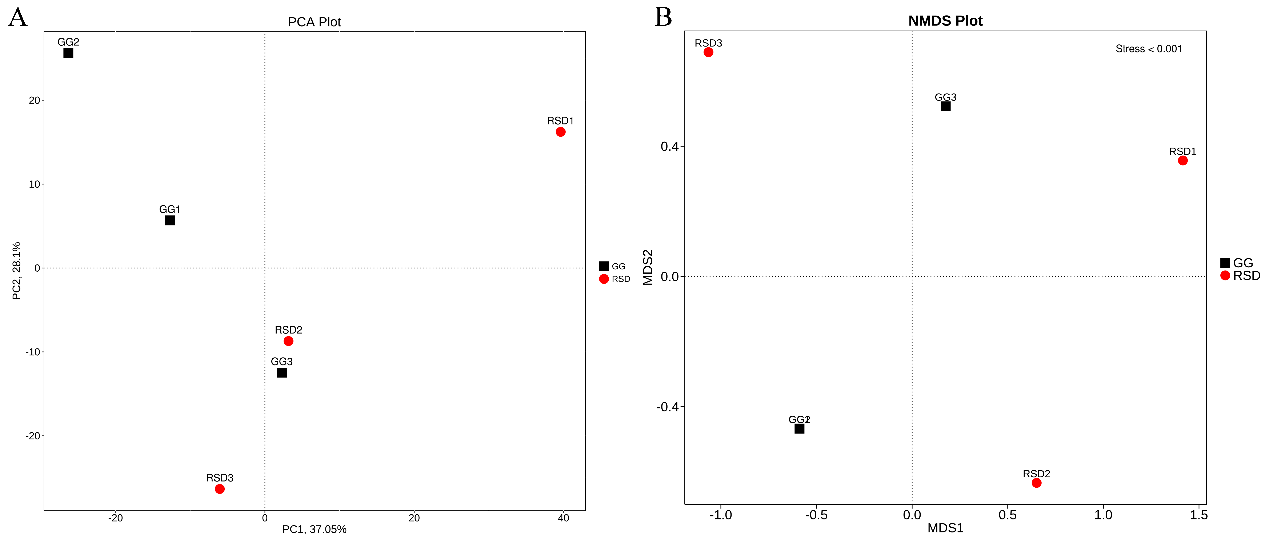


**FIGURE S2.** ANOSIM r-statistics and p-values within- and between- groups at the phylum (A) and genus (B) level. GG refers to the Greylag geese group, RSD refers to the Ruddy shelducks group


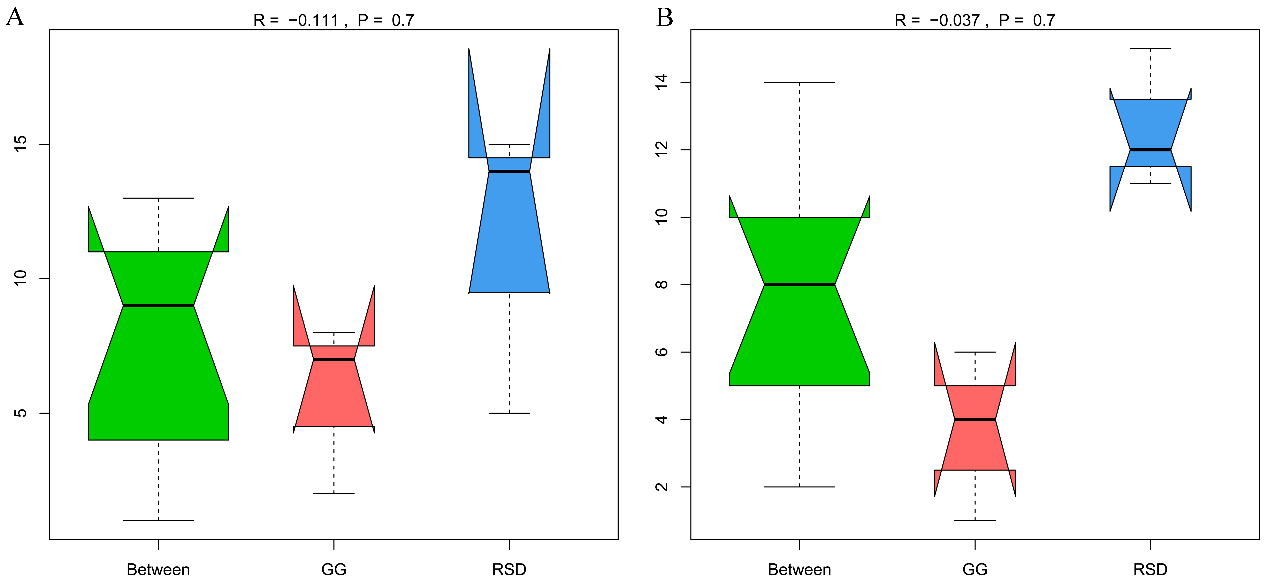


**Table S1.** Summary of the six metagenomes, including sequencing data, assembling data and predicted unigenes data in each sample

|  |  | **GG1** | **GG2** | **GG3** | **RSD1** | **RSD2** | **RSD3** | **Total** | **Average** |
| --- | --- | --- | --- | --- | --- | --- | --- | --- | --- |
| **Sequencing Data** | **Raw Reads (Mb)** | 6,286.50 | 6,213.97 | 6,342.29 | 6,254.97 | 6,799.97 | 6,408.95 | 38,306.65 | 6,384.44 |
|  | **Clean Reads (Mb)** | 6,229.25 | 6,017.01 | 5,770.55 | 6,211.00 | 6,266.14 | 5,979.35 | 36,473.30 | 6078.8833 |
| **Assembling Data** | **Total length of Scaffold (bp)** | 19,959,318 | 98,405,295 | 22,109,202 | 127,712,204 | 25,481,985 | 79,735,533 | - | - |
|  | **Number of Scaffold** | 20,642 | 112,273 | 20,242 | 78,359 | 21,375 | 116,917 | - | - |
|  | **Average length of Scaffold (bp)** | 966.93 | 876.48 | 1,092.24 | 1,629.83 | 1,192.14 | 681.98 | - | - |
|  | **N50 length (bp)** | 922 | 744 | 1,192 | 2,469 | 1,377 | 644 | - | - |
|  | **Maximum length of Scaffold (bp)** | 164,883 | 401,861 | 56,708 | 316,884 | 106,719 | 33,026 | - | - |
|  | **Total length of Scaftig (bp)** | 14,490,857 | 82,326,296 | 18,115,774 | 117,779,170 | 21,840,776 | 66,378,449 | - | - |
|  | **Number of Scaftig** | 14,790 | 91,207 | 17,956 | 75,941 | 18,913 | 100,090 | - | - |
|  | **Average length of Scaftig (bp)** | 979.77 | 902.63 | 1,008.90 | 1,550.93 | 1,154.80 | 663.19 | - | - |
|  | **N50 length (bp)** | 925 | 769 | 1,077 | 2,213 | 1,292 | 628 | - | - |
|  | **Maximum length of Scaftig (bp)** | 163,508 | 401,460 | 28,629 | 268,916 | 86,156 | 22,949 | - | - |
| **Unigenes Prediction** | **Number of unigenes** | 17,169 | 82,101 | 25,362 | 151,477 | 30,197 | 50,011 | - | - |
|  | **Average length of unigenes (bp)** | 468.27 | 501.42 | 536.57 | 688.05 | 568.82 | 240.06 | - | - |
|  | **GC content (%)** | 46.23 | 35.42 | 38.34 | 46.67 | 36.61 | 50.97 | - | - |

**Table S2.** Summary of the number of unigenes used for taxonomic analysis and functional annotation

|  |  | **Number of matched unigenes** | **Ratio** |
| --- | --- | --- | --- |
|  | **Unigenes** | 310,560 | - |
| **Taxonomic Analysis** | **Annotated on NR** | 171,633.00 | 55.27% |
|  | **Annotated on Unclassified** | 13,867.95 | 8.08% |
|  | **Annotated on Kingdom level** | 157,765.05 | 91.92% |
|  | **Annotated on Phylum level** | 150,882.57 | 87.91% |
|  | **Annotated on Class level** | 146,231.32 | 85.20% |
|  | **Annotated on Order level** | 144,926.91 | 84.44% |
|  | **Annotated on Family level** | 132,895.43 | 77.43% |
|  | **Annotated on Genus level** | 128,055.38 | 74.61% |
|  | **Annotated on Species level** | 87,412.69 | 50.93% |
| **Functional Annotation** | **Annotated on KEGG** | 141,279 | 45.49% |
|  | **Annotated on KO** | 82,371 | 26.52% |
|  | **Annotated on KO number** | 7,021 (KOs identified) | - |
|  | **Annotated on pathway** | 49,795 | 16.03% |
|  | **Annotated on pathway number** | 384 (pathways identified) | - |
|  | **Annotated on CAZymes** | 9,060 | 2.92% |
|  | **Annotated on CARD** | 598 | 0.19% |
|  | **Annotated AROs** | 125 (AROs identified) | - |

**Table S3.** Relative abundance of the top four most dominant phyla in each sample

|  | **p__Firmicutes** | **p__Bacteroidetes** | **p__Proteobacteria** | **p__Tenericutes** | **p__Fusobacteria** | **Others** |
| --- | --- | --- | --- | --- | --- | --- |
| **GG1** | 14.23% | 0.59% | 13.81% | 5.54% | 1.00% | 60.84% |
| **GG2** | 5.76% | 0.32% | 16.59% | 13.45% | 1.76% | 60.11% |
| **GG3** | 74.94% | 0.26% | 4.13% | 0.05% | 0.11% | 18.86% |
| **Average** | 31.64% | - | 11.51% | 6.34% | 0.96% | 46.60% |
| **RSD1** | 33.82% | 46.69% | 1.35% | 0.07% | 4.93% | 11.86% |
| **RSD2** | 81.14% | 0.12% | 1.58% | 0.45% | 5.25% | 11.19% |
| **RSD3** | 5.00% | 0.17% | 8.85% | 0.00% | 0.01% | 83.09% |
| **Average** | 39.99% | 15.66% | 3.93% | - | 3.40% | 35.38% |

**Table S4.** Relative abundance of the top five most dominant genera in each sample

|  | **GG1** | **GG2** | **GG3** | **Average** | **RSD1** | **RSD2** | **RSD3** | **Average** |
| --- | --- | --- | --- | --- | --- | --- | --- | --- |
| **g__Streptococcus** | 2.23% | 1.69% | 17.83% | 7.25% | - | - | - | - |
| **g__Escherichia** | 7.35% | 9.45% | 0.36% | 5.72% | - | - | - | - |
| **g__Mycoplasma** | 4.69% | 11.31% | 0.03% | 5.34% | - | - | - | - |
| **g__Romboutsia** | 0.00% | 0.00% | 15.81% | 5.27% | - | - | - | - |
| **g__Staphylococcus** | 6.58% | 0.13% | 1.09% | 2.60% | - | - | - | - |
| **g__Enterococcus** | - | - | - | - | 0.04% | 49.97% | 0.08% | 16.70% |
| **g__Bacteroides** | - | - | - | - | 32.60% | 0.01% | 0.01% | 10.87% |
| **g__Streptococcus** | - | - | - | - | 1.01% | 18.92% | 1.57% | 7.17% |
| **g__Megamonas** | - | - | - | - | 6.64% | 0.01% | 0.00% | 2.22% |
| **g__Lactobacillus** | - | - | - | - | 0.05% | 5.07% | 0.43% | 1.85% |
| **Others** | 75.06% | 75.56% | 57.39% | 69.34% | 54.34% | 25.52% | 91.87% | 57.24% |

**Table S5.** The differentially abundant genera detected by Metastats analysis. A p value less than 0.05 was considered statistically significant

| **Group** | **Taxa** | **p value** |
| --- | --- | --- |
| **Higher in GG group** | g__Pichia | 0.0487 |
|  | g__Phlebia | 0.0484 |
|  | g__Halorhabdus | 0.0472 |
|  | g__Ferroglobus | 0.0467 |
|  | g__Neochlamydia | 0.0461 |
|  | g__Polynucleobacter | 0.0447 |
|  | g__Thermococcus | 0.0444 |
|  | g__Porphyrobacter | 0.0435 |
|  | g__Andreprevotia | 0.0420 |
|  | g__Halobacteroides | 0.0397 |
|  | g__Fibulorhizoctonia | 0.0392 |
|  | g__Pelagirhabdus | 0.0382 |
|  | g__Massilia | 0.0376 |
|  | g__Eremococcus | 0.0374 |
|  | g__Campylobacter | 0.0369 |
|  | g__Aureobasidium | 0.0359 |
|  | g__Sneathia | 0.0329 |
|  | g__Varibaculum | 0.0326 |
|  | g__Coxiella | 0.0321 |
|  | g__Shigella | 0.0318 |
|  | g__Cronobacter | 0.0309 |
|  | g__Ruegeria | 0.0307 |
|  | g__Hydrogenobaculum | 0.0298 |
|  | g__T4virus | 0.0289 |
|  | g__Atopococcus | 0.0280 |
|  | g__Pseudorhodoferax | 0.0273 |
|  | g__Magnaporthiopsis | 0.0269 |
|  | g__Pseudopedobacter | 0.0266 |
|  | g__Methanobrevibacter | 0.0236 |
|  | g__Methylobacterium | 0.0234 |
|  | g__Absidia | 0.0180 |
|  | g__Microcystis | 0.0171 |
|  | g__Magnaporthe | 0.0171 |
|  | g__Leptotrichia | 0.0127 |
|  | g__Lodderomyces | 0.0109 |
|  | g__Candida | 0.0093 |
|  | g__Agaricus | 0.0088 |
|  | g__Kalmanozyma | 0.0086 |
|  | g__Moesziomyces | 0.0085 |
|  | g__Lambdavirus | 0.0069 |
|  | g__Talaromyces | 0.0064 |
| **Higher in RSD group** | g__Eubacterium | 0.0486 |
|  | g__Holdemanella | 0.0458 |
|  | g__Thermoanaerobacterium | 0.0455 |
|  | g__Aliivibrio | 0.0437 |
|  | g__Thiothrix | 0.0339 |
|  | g__Arcanobacterium | 0.0302 |
|  | g__Gardnerella | 0.0277 |
|  | g__Clostridiisalibacter | 0.0262 |
|  | g__Macrophomina | 0.0254 |
|  | g__Peptoclostridium | 0.0220 |
|  | g__Sebaldella | 0.0141 |
|  | g__Nitratiruptor | 0.0063 |

**Table S6.** Significantly different KEGG pathways (level 3) between GG and RSD group using Metastats analysis. A p value less than 0.05 was considered statistically significant

| **Group** | **KEGG pathway (level 3)** | **Pathway Name** | **Class** | **p value** |
| --- | --- | --- | --- | --- |
| **Higher in RSD group** | ko00040 | Pentose and glucuronate interconversions | Carbohydrate metabolism | 0.0279 |
|  | ko03013 | RNA transport | Translation | 0.0273 |
|  | ko03320 | PPAR signaling pathway | Endocrine system | 0.0265 |
|  | ko04146 | Peroxisome | Transport and catabolism | 0.0084 |
| **Higher in GG group** | ko00240 | Pyrimidine metabolism | Nucleotide metabolism | 0.0456 |
|  | ko00903 | Limonene and pinene degradation | Metabolism of terpenoids and polyketides | 0.0451 |
|  | ko00625 | Chloroalkane and chloroalkene degradation | Xenobiotics biodegradation and metabolism | 0.0361 |
|  | ko00626 | Naphthalene degradation | Xenobiotics biodegradation and metabolism | 0.0200 |
|  | ko00906 | Carotenoid biosynthesis | Metabolism of terpenoids and polyketides | 0.0113 |
